# Characterization of the Quality Factor Due to the Static Prestress in Classical Caputo and Caputo–Fabrizio Fractional Thermoelastic Silicon Microbeam

**DOI:** 10.3390/polym13010027

**Published:** 2020-12-23

**Authors:** Hamdy M. Youssef, Alaa A. El-Bary, Eman A. N. Al-Lehaibi

**Affiliations:** 1 Mechanical Engineering Department, College of Engineering and Islamic Architecture, Umm Al-Qura University, Makkah 21955, Saudi Arabia; 2 Mathematics Department, Faculty of Education, Alexandria University, 21500 Alexandria, Egypt; 3 Arab Academy for Science, Technology and Maritime Transport, P.O. Box 1029 Alexandria, Egypt; aaelbary@aast.edu; 4 National Committee for Mathematics, Academy of Scientific Research and Technology, 11516 Cairo, Egypt; 5 Mathematics Department, Al-Lith University College, Umm Al-Qura University, Al-Lith 28433, Saudi Arabia; dremanallehaibi@gmail.com

**Keywords:** static prestress, microresonator, Lord–Shulman, dual-phase lag, quality factor, fractional derivative, Caputo–Fabrizio

## Abstract

The thermal quality factor is the most significant parameter of the micro/nanobeam resonator. Less energy is released by vibration and low damping, which results in greater efficiency. Thus, for a simply supported microbeam resonator made of silicon (Si), a thermal analysis of the thermal quality factor was introduced. A force due to static prestress was considered. The governing equations were constructed in a unified system. This system generates six different models of heat conduction; the traditional Lord–Shulman, Lord–Shulman based on classical Caputo fractional derivative, Lord–Shulman based on the Caputo–Fabrizio fractional derivative, traditional Tzou, Tzou based on the classical Caputo fractional derivative, and Tzou based on the Caputo–Fabrizio fractional derivative. The results show that the force due to static prestress, the fractional order parameter, the isothermal value of natural frequency, and the beam’s length significantly affect the thermal quality factor. The two types of fractional derivatives applied have different and significant effects on the thermal quality factor.

## 1. Introduction

Several applications are essential for micro-and nanoelectric resonator applications, such as mechanical signal processing, scanning, microscopes, and ultrasensitive mass detection. The energy dissipation mechanism for improving micro/nanoelectric resonators is being investigated [1,2,3,4]. Zener has introduced and given an approximately analytical form of the heat quality factor in thermoelastic dissipation. In the handling of viscothermoelastic, Zener also explored thermoelastic damping in beams [5,6,7]. Many scholars have performed various studies on the classic Fourier heat conduction rule for thermoelastic damp. Lifshitz and Roukes [8] gave an exact expression for thermoelastic damping. Sun et al. [9] studied the thermoelastic damping of a beam resonator based on a non-Fourier heat conduction equation. Sharma and Sharma [10] introduced the thermal damping in a circular plate of microresonators in the context of the Lord–Shulman theory of generalized thermoelasticity theory (L–S).

Tzou introduced the dual-phase-lag heat conduction (DPL) in which the temperature gradient and heat flux lag times have been included [11,12]. Many scientists and authors have applied this model to heat transfer problems [13], physical systems [14,15,16,17,18], and thermoelastic damping vibration [19,20]. Guo et al. [21,22] studied the thermoelastic damping theory of micro- and nanomechanical resonators using the DPL model. The DPL is used in mathematics, physics and many other scientific fields, such as applied mechanics, chemicals, biology, etc., of a fractional derivative definition [23,24,25,26]. The fractional derivative is a powerful way to clarify the history and patrimony of different materials and processes. The definition of fractional derivatives was found to be more applicable to real-world modeling applications than the regular integer derived [27,28,29]. Several scholars have concentrated more on establishing or producing a new concept of fractional derivative, such as the Riemann–Liouville and the Caputo, [30,31,32]. Youssef developed a theory of thermoelasticity based on fractional heat conductivity and other thermoelasticity theories [33,34]. Sherif et al. introduced a different theory of thermoelasticity using the methodology of fractional calculus [35]. The fractional calculus models are more consistent than standard ones since they predict delayed effects. Some researchers realized that new fractional derivatives could satisfy the need with some special or nonsingular kernels by providing an exponential solution to the single kernel issue found in the Liouville–Caputo, Riemann–Liouville, Caputo and Fabrizio fractional derivatives concepts [36,37].

Small size leads to an increase in the natural frequency of the thermoelastic resonator. The resonators’ normal frequency can also be influenced by an axial force like static stress [3,38]. The natural frequency decreases the pressure power, while the lower frequency increases the energy of the tensile. For experimental tests, the frequency change of the tuning resonators was used [3,39].

Therefore, in the present study, we will construct an analytical solution of a thermal model of a silicon microbeam resonator subjected to static prestress based on two different types of fractional derivative definitions. We will calculate the thermal quality factor in the context of the new Caputo-Fabrizio fractional derivative which will supply us with new and different results. The governing equations will be constructed in the context of Lord–Shulman and dual-phase-lag heat conduction models. The thermal quality factor will be introduced and calculated for different cases and various values of parameters. This paper introduces not only different and new comparisons between the Lord–Shulman (L-S) and dual-phase-lag (DPL) models but also between two types of fractional derivative and the traditional derivative which has not been published before this work.

## 2. Model Formulation

Tzou proposed a dual-phase lag with two distinct relaxation time parameters in the conventional thermal flow model and adherence to the effect of micro/nanostructure interactions [40,41,42,43]:

(1) $$-K\nabla T\left(x,t+\tau_{T}\right)=q\left(x,t+\tau_{q}\right)$$

where T is the temperature, K is the heat conductivity, q is the heat flux, and t is the time, $\tau_{q},\tau_{T}$ are the phase lag of the heat flux and the phase lag of the temperature gradient, respectively. 

Expand the two sides of the Equation (1) considering the fractional-order definition to obtain:

(2) $$-K\left[\nabla T\left(x,t\right)+\tau_{T}^{\beta }D_{t}^{\beta }\nabla T\left(x,t\right)\right]=q\left(x,t\right)+\tau_{q}^{\beta }D_{t}^{\beta }q\left(x,t\right)$$

The classical Caputo and Caputo–Fabrizio define the fractional derivative operator $D_{t}^{\beta }=\frac{d^{\beta }}{dt^{\beta }}$ in the equation as follows [34,36,44,45,46,47]:

(3) $$D_{t}^{\beta }f\left(t\right)=\left\{\begin{array}{ccc} \frac{1}{\Gamma \left(1-\beta \right)}\int_{0}^{t}f^{\prime }\left(\xi \right){\left(t-\xi \right)}^{-\beta }\;d\xi & 0\leq \beta <1 & \mathrm{Classical}\;\mathrm{Caputo}\\ \frac{1}{1-\beta }\int_{0}^{t}f^{\prime }\left(\xi \right)\exp\left(-\frac{\beta }{1-\beta }\left(t-\xi \right)\right)\;d\xi & 0\leq \beta <1 & \mathrm{Caputo}-\mathrm{Fabrizio}\\ f^{\prime }\left(t\right) & \beta =1 & \mathrm{Normal}\;\mathrm{derivative} \end{array}\right\}$$

**Figure 1:**
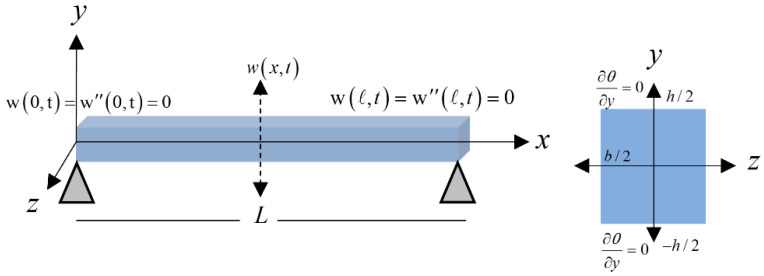
Nanobeam of silicon.

The constitutive equations of Euler–Bernoulli’s microbeam with length L, width b and depth h along the *x*, *y*, and *z*-axes, respectively, have been considered in Figure 1. The moment of inertia is *I*, and applied axial force is F, and thermoelastic displacement (lateral deflection) is w(x,t) as [9,10,11,12]:

(4) $$\sigma_{xx}\left(x,y,z,t\right)=\sigma_{0}-Ey\frac{\partial^{2}w\left(x,t\right)}{\partial x^{2}}-E\alpha_{T}\theta \left(x,y,z,t\right)$$

where $\sigma_{0}=\frac{F}{bh}$ is the stress due to the applied axial force, E is the Young’s Modulus, and $\alpha_{T}$ is defined as the coefficient of thermal expansion. The reference temperature of the beam is $T_{0}$, and the temperature increment is $\theta =\left(T-T_{0}\right)$.

The equation of motion along the beam’s length is [12,48,49,50]:

(5) $$\frac{\partial^{4}w\left(x,t\right)}{\partial x^{4}}-\frac{F}{EI}\frac{\partial^{2}w\left(x,t\right)}{\partial x^{2}}+\frac{\alpha_{T}}{I}\frac{\partial^{2}I_{T}\left(x,y,z,t\right)}{\partial x^{2}}+\frac{\rho A}{EI}\frac{\partial^{2}w\left(x,t\right)}{\partial t^{2}}=0$$

where

(6) $$I\left(x,y,z,t\right)=\int_{-b/2}^{b/2}\int_{-h/2}^{h/2}y^{2}dydz=\frac{bh^{3}}{12}$$

and

(7) $$I_{T}\left(x,y,z,t\right)=\;\int_{-b/2}^{b/2}\int_{-h/2}^{h/2}y\;\theta \left(x,y,z,t\right)\;dy\;dz$$

Equation (5) takes the form

(8) $$\frac{\partial^{4}w\left(x,t\right)}{\partial x^{4}}-\frac{12F}{Ebh^{3}}\frac{\partial^{2}w\left(x,t\right)}{\partial x^{2}}+\frac{12\alpha_{T}}{h^{3}}\frac{\partial^{2}}{\partial x^{2}}\;\int_{-h/2}^{h/2}y\;\theta \left(x,y,z,t\right)\;dy\;+\frac{12\rho }{Eh^{2}}\frac{\partial^{2}w\left(x,t\right)}{\partial t^{2}}=0$$

The fractional dual-phase-lag heat equations take the form [19,20,51]:

(9) $$\left(1+\tau_{T}^{\beta }D_{t}^{\beta }\right)\nabla^{2}\theta \left(x,y,z,t\right)=\frac{\partial }{\partial t}\left(1+\tau_{q}^{\beta }D_{t}^{\beta }\right)\left(\frac{\rho \;C_{\nu }}{K}\theta \left(x,y,z,t\right)+\frac{E\alpha_{T}T_{0}}{K\left(1-2\nu \right)}\varepsilon \left(x,y,z,t\right)\right)$$

where υ is the Poisson’s ratio and $C_{\nu }$ is the specific heat at constant strain.

The volumetric deformations are as follows:

(10) $$\varepsilon =\varepsilon_{xx}+\varepsilon_{yy}+\varepsilon_{zz}$$

where

(11) $$E\varepsilon_{xx}\left(x,y,z,t\right)=\sigma_{0}-yE\frac{\partial^{2}w\left(x,t\right)}{\partial x^{2}}\;$$

and

(12) $$E\varepsilon_{yy}\left(x,y,z,t\right)=E\varepsilon_{zz}\left(x,y,z,t\right)=-\upsilon \sigma_{0}+\upsilon yE\frac{\partial^{2}w\left(x,t\right)}{\partial x^{2}}+\left(1+\upsilon \right)\alpha_{T}E\theta \left(x,y,z,t\right)$$

By making sum the strain components in (12), we obtain

(13) $$E\varepsilon \left(x,y,z,t\right)=\left(1-2\upsilon \right)\sigma_{0}-\left(1-2\upsilon \right)yE\frac{\partial^{2}w\left(x,t\right)}{\partial x^{2}}+2\left(1+\upsilon \right)\alpha_{T}E\theta \left(x,y,z,t\right)$$

Substituting Equation (13) into the Equation (9), we obtain

(14) $$\begin{array}{c} \left(1+\tau_{T}^{\beta }D_{t}^{\beta }\right)\nabla^{2}\theta \left(x,y,z,t\right)=\eta \frac{\partial }{\partial t}\left(1+\tau_{q}^{\beta }D_{t}^{\beta }\right)\theta \left(x,y,z,t\right)+\;\\ \frac{\alpha_{T}T_{0}}{K}\frac{\partial }{\partial t}\left(1+\tau_{q}^{\beta }D_{t}^{\beta }\right)\left[2\frac{\left(1+\upsilon \right)\alpha_{T}}{\left(1-2\upsilon \right)}E\theta \left(x,y,z,t\right)-yE\frac{\partial^{2}w\left(x,t\right)}{\partial x^{2}}\right] \end{array}$$

which gives:

(15) $$\begin{array}{c} \left(1+\tau_{T}D_{t}^{\beta }\right)\nabla^{2}\theta \left(x,y,z,t\right)+\frac{\alpha_{T}T_{0}E}{K}y\frac{\partial }{\partial t}\left(1+\tau_{q}D_{t}^{\beta }\right)\frac{\partial^{2}w\left(x,t\right)}{\partial x^{2}}=\\ \frac{\partial }{\partial t}\left(1+\tau_{q}D_{t}^{\beta }\right)\left(\eta +\frac{2ET_{0}\alpha_{T}^{2}\left(1+\upsilon \right)}{K\left(1-2\nu \right)}\right)\theta \left(x,y,z,t\right) \end{array}$$

where $\eta =\frac{\rho C_{\upsilon }}{K}$.

Hence, the Equation (15) takes the form:

(16) $$\begin{array}{c} \left(1+\tau_{T}^{\beta }D_{t}^{\beta }\right)\nabla^{2}\theta \left(x,y,z,t\right)+\frac{\Delta_{E}}{\alpha_{T}}\frac{\partial }{\partial t}\left(1+\tau_{q}^{\beta }D_{t}^{\beta }\right)y\frac{\partial^{2}w\left(x,t\right)}{\partial x^{2}}=\\ \frac{\partial }{\partial t}\left(1+\tau_{q}^{\beta }D_{t}^{\beta }\right)\left(\eta +2\Delta_{E}\frac{\left(1+\upsilon \right)}{\left(1-2\upsilon \right)}\right)\theta \left(x,y,z,t\right) \end{array}$$

where $\Delta_{E}=\frac{T_{0}\alpha_{T}^{2}E_{0}}{K}$.

Because no gradient exists in the z-direction, then, $\nabla^{2}\theta \left(x,y,z,t\right)\approx \frac{\partial^{2}\theta \left(x,y,t\right)}{\partial y^{2}}$, therefore, we obtain

(17) $$\frac{\partial^{4}w\left(x,t\right)}{\partial x^{4}}-\frac{12F}{Ebh^{3}}\frac{\partial^{2}w\left(x,t\right)}{\partial x^{2}}+\frac{12\alpha_{T}}{h^{3}}\frac{\partial^{2}}{\partial x^{2}}\;\int_{-h/2}^{h/2}y\;\theta \left(x,y,t\right)\;dy\;+\frac{12\rho }{Eh^{2}}\frac{\partial^{2}w\left(x,t\right)}{\partial t^{2}}=0$$

(18) $$\begin{array}{c} \left(1+\tau_{T}^{\beta }D_{t}^{\beta }\right)\frac{\partial^{2}\theta \left(x,y,t\right)}{\partial y^{2}}+\frac{\Delta_{E}}{\alpha_{T}}y\;\frac{\partial }{\partial t}\left(1+\tau_{q}^{\beta }D_{t}^{\beta }\right)\frac{\partial^{2}w\left(x,t\right)}{\partial x^{2}}=\\ \frac{\partial }{\partial t}\left(1+\tau_{q}^{\beta }D_{t}^{\beta }\right)\left(\eta +2\Delta_{E}\frac{\left(1+\upsilon \right)}{\left(1-2\upsilon \right)}\right)\theta \left(x,y,t\right) \end{array}$$

We consider the following functions:

(19) $$w(x,t)=W(x)\;e^{i\omega t},\;\theta (x,y,t)=\vartheta (x,y)\;e^{i\omega t}$$

where ω is called the isothermal frequency parameter, and $i=\sqrt{-1}$.

Hence, Equations (17) and (18) will take the forms

(20) $$\frac{d^{4}W\left(x\right)}{dx^{4}}-\frac{12F}{Ebh^{3}}\frac{d^{2}W\left(x\right)}{dx^{2}}+\frac{12\alpha_{T}}{h^{3}}\frac{d^{2}}{dx^{2}}\;\int_{-h/2}^{h/2}y\;\;\vartheta \left(x,y\right)dy\;-\frac{12\rho \omega^{2}}{Eh^{2}}W\left(x\right)=0$$

and

(21) $$\begin{array}{c} \frac{\partial^{2}\vartheta \left(x,y\right)}{\partial y^{2}}+\frac{\Delta_{E}i\omega \left(1+\tau_{q}^{\beta }\delta \left(\omega ,\beta \right)\right)}{\alpha_{T}\left(1+\tau_{T}^{\beta }\delta \left(\omega ,\beta \right)\right)}y\frac{d^{2}W\left(x\right)}{dx^{2}}=\\ \frac{i\omega \left(1+\tau_{q}^{\beta }\delta \left(\omega ,\beta \right)\right)}{\left(1+\tau_{T}^{\beta }\delta \left(\omega ,\beta \right)\right)}\left(\eta +2\Delta_{E}\frac{\left(1+\upsilon \right)}{\left(1-2\upsilon \right)}\right)\vartheta \left(x,y\right) \end{array}$$

Through simplifying Equation (21), we applied the following relations [26,35,52,53]:

(22) $$D_{t}^{\beta }\left(e^{i\omega t}\right)=\delta \left(\omega ,\beta \right)e^{i\omega t}$$

where

(23) $$\delta \left(\omega ,\beta \right)=\left\{\begin{array}{cc} \delta_{1}={\left(i\omega \right)}^{\beta } & \mathrm{Classical}-\mathrm{Caputo}\;\left(C-C\right)\\ \delta_{2}=\frac{i\omega \;}{\beta -i\omega \left(\beta -1\right)} & \mathrm{Fabrizio}-\mathrm{Caputo}\;\left(F-C\right)\\ \delta_{3}=i\omega & \mathrm{Traditional}\;\mathrm{Derivative} \end{array}\right\}$$

## 3. Descriptions of the Six Models 

To describe the six different models, we follow the following substitutions:

1. $\beta =1.0,\;\tau_{T}=0.0,\;\tau_{q}=\tau_{0}$, and $\;\delta =\delta_{3}$ represents the traditional Lord–Shulman model with one relaxation time [54].
2. $0.0\leq \beta <1.0,\;\tau_{T}=0.0,\;\tau_{q}=\tau_{0}$, and $\delta =\delta_{1}$ represents the Lord–Shulman model based on the classical Caputo fractional definition (Lord–Shulman (C-C)).
3. $0.0\leq \beta <1.0,\;\tau_{T}=0.0,\;\tau_{q}=\tau_{0},\;\mathrm{and}\;\;\delta =\delta_{2}$ represents the Lord–Shulman model based on the Caputo–Fabrizio fractional definition (Lord–Shulman (F-C)).
4. $\beta =1.0,\;\tau_{T}\neq \;\tau_{q}\neq 0.0$, and $\delta =\delta_{3}$ represents the traditional Tzou dual-phase-lag (DPL) model.
5. $0.0\leq \beta <1.0,\;\;\tau_{T}\neq \;\tau_{q}\neq 0.0$, and$\delta =\delta_{1}$ represents the Tzou dual-phase-lag (DPL) model based on the classical Caputo fractional definition (Tzou (C-C)) [42].
6. $0.0\leq \beta <1.0,\;\tau_{T}\neq \;\tau_{q}\neq 0.0,\;\mathrm{and}\;\delta =\delta_{2}$ represents the Tzou dual-phase-lag (DPL) model based on the Caputo–Fabrizio fractional definition (Tzou (F-C)).

Equation (21) could be written in the form

(24) $$\frac{\partial^{2}\vartheta \left(x,y\right)}{\partial y^{2}}-\;\;\lambda^{2}\vartheta \left(x,y\right)=-\alpha y\frac{d^{2}W\left(x\right)}{dx^{2}}$$

where $\lambda^{2}=\frac{i\omega \left(1+\tau_{q}^{\beta }\delta \left(\omega ,\beta \right)\right)}{\left(1+\tau_{T}^{\beta }\delta \left(\omega ,\beta \right)\right)}\left(\eta +2\Delta_{E}\frac{\left(1+\upsilon \right)}{\left(1-2\upsilon \right)}\right)$ and $\alpha =\frac{\Delta_{E}i\omega \left(1+\tau_{q}^{\beta }\delta \left(\omega ,\beta \right)\right)}{\alpha_{T}\left(1+\tau_{T}^{\beta }\delta \left(\omega ,\beta \right)\right)}$.

The general solution of the differential Equation (23) takes the form:

(25) $$\vartheta \left(x,y\right)=A\cos\left(\lambda y\right)+B\sin\left(\lambda y\right)+\frac{\alpha }{\lambda^{2}}y\frac{d^{2}W\left(x\right)}{dx^{2}}$$

The boundary conditions are as follows:

(26) $${\frac{\partial \vartheta \left(x,y\right)}{\partial y}|}_{y=\pm h/2}=0$$

Hence, we obtain

(27) $$\vartheta \left(x,y\right)=\left[y-\frac{\sin\left(\lambda y\right)}{\lambda \cos\left(\lambda h/2\right)}\right]\frac{\alpha }{\lambda^{2}}\frac{d^{2}W\left(x\right)}{dx^{2}}$$

From Equations (5), (7), and (19), we obtain

(28) $$\frac{h}{12\rho }Ef\left(\omega \right)\frac{d^{4}W\left(x\right)}{dx^{4}}-\frac{E}{\rho bh^{2}}F\frac{d^{2}W\left(x\right)}{dx^{2}}=\omega^{2}W\;\left(x\right)\;$$

where

(29) $$f\left(\omega \right)=1+\frac{\alpha_{T}b\alpha }{\lambda^{2}I}\left(\frac{h^{3}}{12}+\frac{h}{\lambda^{2}}-\frac{2}{\lambda^{3}}\tan\left(\lambda h/2\right)\right)$$

Hence, we have

(30) $$\frac{IE_{\omega }}{\rho A}\left(\frac{d^{4}W\left(x\right)}{dx^{4}}-\frac{F}{IE_{\omega }}\frac{d^{2}W\left(x\right)}{dx^{2}}\right)=\omega^{2}W\left(x\right)$$

and

(31) $$E_{\omega }=E\;f\left(\omega \right)$$

For a simply supported beam, the exact analytical solution for the natural frequency is given as [9,10,38]:

(32) $$\omega =\frac{\pi }{L\sqrt{\rho A}}\sqrt{\frac{\pi^{2}IE}{L^{2}}\;f\left(\omega_{0}\right)+F}$$

where $\omega_{0}$ is the isothermal value of frequency given by [38]:

(33) $$\omega_{0}=q_{n}^{2}h\sqrt{\frac{E}{12\rho }},\;q_{n}\approx \frac{1}{L}\left\{4.73,7.853,10.996,\ldots \right\},\;n=1,2,3,\ldots$$

Hence, we get

(34) $$\omega =\frac{\pi^{2}}{L^{2}}\sqrt{\frac{I\;E}{\rho A}}\sqrt{\left(1+\frac{\Delta E\;b\;h^{3}}{12I\;\varepsilon_{1}}\left(1+\frac{12\;\varepsilon 2}{h^{2}\varepsilon 1}-\frac{24}{h^{3}}\tan\left(\lambda h/2\right){\left(\frac{\varepsilon_{1}}{1+\tau_{T}\delta \left(\omega ,\beta \right)}\right)}^{-3/2}\right)\right)+\frac{L^{2}}{\pi^{2}IE}F}$$

where $\varepsilon_{1}=\eta +\frac{2\Delta E\left(1+\upsilon \right)}{1-2\upsilon },\;\varepsilon_{2}=\frac{1+\tau_{T}^{\beta }\delta \left(\omega ,\beta \right)}{i\;\omega_{0}\left(1+\tau_{q}^{\beta }\delta \left(\omega ,\beta \right)\right)}$.

The thermal quality factor $\left(\frac{1}{Q}\right)$ is defined as the quality of the thermal damping and defined as [4,5,6,7,55,56]:

(35) 1Q=2|Im(ω)Re(ω)|

## 4. Numerical Results and Discussions

The relation between the variation of the thermoelastic damping and the beam height h, and the beam length L in different values of the applied axial force F on the microbeam will be studied. Simple clamping at the two ends of the microbeam resonator made of silicon will be explored. Hence, the material properties of silicon (Si) will be taken as follows [9]:

$$\begin{array}{l} T_{0}=300\left(K\right),\;\rho =2330\left(\mathrm{kg}\;m^{-3}\right),\;K=141.04\;\left({\mathrm{Wm}}^{-1}k^{-1}\right),\;C_{\upsilon }=1.64\times 10^{6}\;\left({\mathrm{Jm}}^{-3}K^{-1}\right),\;E=165\;\left(\mathrm{GPa}\right)\\ \alpha_{T}=2.6\times 10^{-6}\left(K^{-1}\right),\;\tau_{T}=5.0\times 10^{-7}\left(s\right),\;\tau_{q}=5.0\times 10^{-6}\left(s\right),\;\tau_{0}=5.0\times 10^{-10}\left(s\right) \end{array}$$

The aspect ratios of the beam are assumed L=50×h and b=4×h. For the microscale beam, we will take the beam’s thickness $h=1.0\times 10^{-7}\;\left(m\right)$.

**Figure 2:**
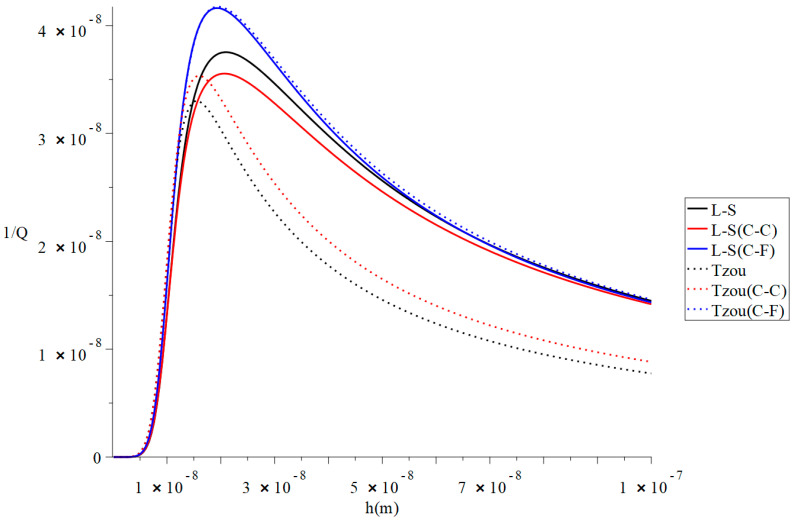
The thermal quality factor is based on the Lord-Shulman (L-S) and Tzou models with various fractional derivative types $\beta =0.8,\;\;L=50\;h,\;\;b=4h,\;\;q=4.73/L,\;\;\mathrm{and}\;F=10^{-40}$.

**Figure 3:**
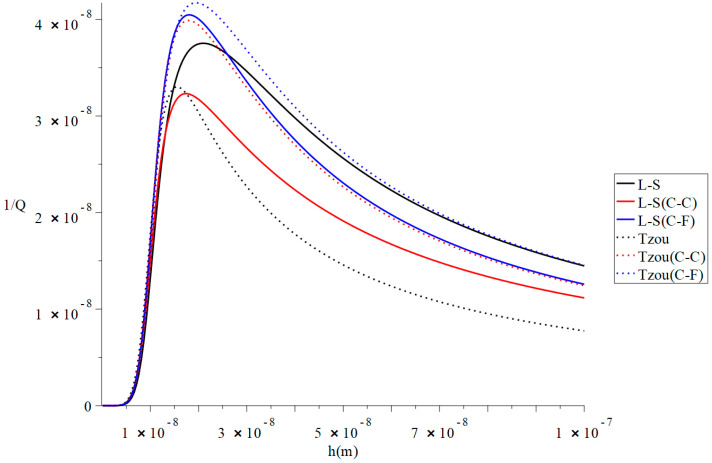
The thermal quality factor is based on the Lord-Shulman (L-S) and Tzou models with various fractional derivative types $\beta =0.3,\;\;L=50\;h,\;\;b=4h,\;\;q=4.73/L,\;\;\mathrm{and}\;F=10^{-40}$.

Figure 2 and Figure 3 represent the thermal quality factor $\left(\frac{1}{Q}\right)$ in the context of Lord–Shulman and Tzou models based on the classical case when β=1.0, classical Caputo (C-C), and Caputo–Fabrizio (F-C) fractional derivative definitions, respectively. We considered two different values of the fractional-order parameter β=(0.8,0.3) when $L=50\;\;h,\;\;b=4\;h,\;\;q=4.73/L,\;\;\;\mathrm{and}\;F=10^{-40}$ to stand on its effect on the thermal quality factor.

Figure 2 shows that the type of the fractional derivative and the values of the fractional-order parameter β have significant effects on the thermal quality factor in the context of the Lord–Shulman and Tzou models. The thermal quality factor values are close in the context of Caputo–Fabrizio based on Lord–Shulman and Tzou models. The peak points are in the following order:

(36) $$Q_{\mathrm{Max}}^{-1}\left(\mathrm{Tozu}\left(C-F\right)\right)>Q_{\mathrm{Max}}^{-1}\left(L-S\left(C-F\right)\right)>Q_{\mathrm{Max}}^{-1}\left(L-S\right)>Q_{\mathrm{Max}}^{-1}\left(\mathrm{Tozu}\left(C-C\right)\right)>Q_{\mathrm{Max}}^{-1}\left(L-S\left(C-C\right)\right)>Q_{\mathrm{Max}}^{-1}\left(\mathrm{Tozu}\right)$$

Figure 3 shows that when β=1.0 and β=0.3, the values of the thermal quality factor are not close in the context of Caputo–Fabrizio based on the Lord–Shulman and Tzou models, as in Figure 1. Moreover, a change in the fractional-order parameter value leads to a change in the values of the six models’ peak points. The peak points are in the following order:

(37) $$Q_{\mathrm{Max}}^{-1}\left(\mathrm{Tozu}\left(C-F\right)\right)>Q_{\mathrm{Max}}^{-1}\left(L-S\left(C-F\right)\right)>Q_{\mathrm{Max}}^{-1}\left(\mathrm{Tozu}\left(C-C\right)\right)>Q_{\mathrm{Max}}^{-1}\left(L-S\right)>Q_{\mathrm{Max}}^{-1}\left(\mathrm{Tozu}\right)>Q_{\mathrm{Max}}^{-1}\left(L-S\left(C-C\right)\right)$$

**Figure 4:**
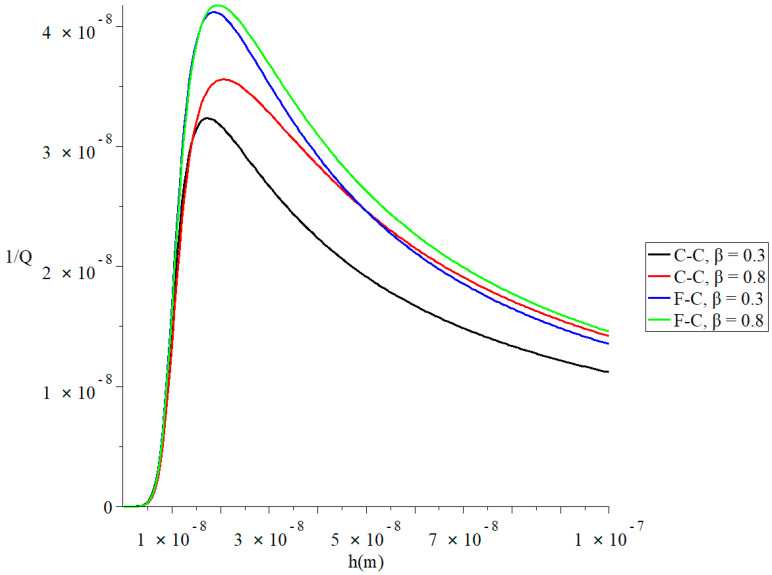
The thermal quality factor is based on the Lord-Shulman model with various fractional derivative types when $L=50\;h,\;\;b=4h,\;\;q=4.73/L,\;\;\mathrm{and}\;F=10^{-40}$.

**Figure 5:**
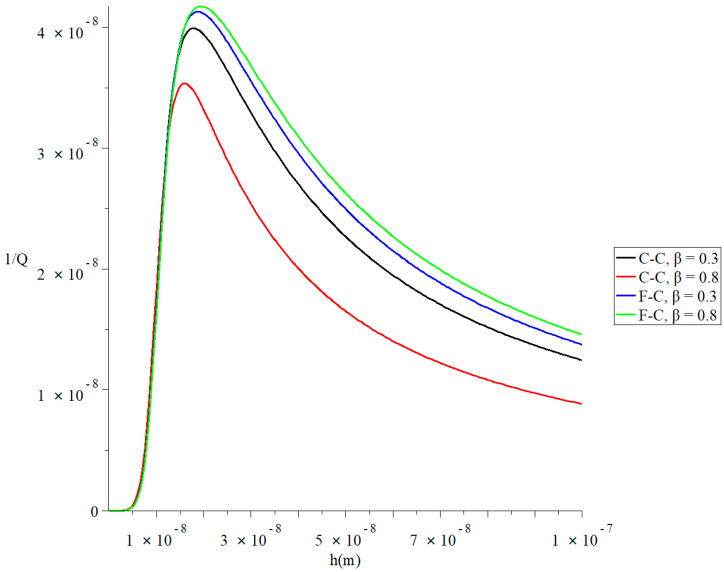
The thermal quality factor is based on the Tzou model with various fractional derivative types when $L=50\;h,\;\;b=4h,\;\;q=4.73/L,\;\;\mathrm{and}\;F=10^{-40}$.

Figure 4 and Figure 5 represent the thermal quality factor $\left(\frac{1}{Q}\right)$ in the context of Lord–Shulman and Tzou models based on the classical Caputo (C-C) and Caputo–Fabrizio (F-C) factional derivative definitions, respectively. We considered two different values of the fractional-order parameter β=(0.3,0.8) when $L=50\;h,\;b=4h,\;q=4.73/L,\;\mathrm{and}\;F=10^{-40}$ to stand on its effect on the thermal quality factor.

Figure 4 shows that the type of the fractional derivative and the fractional-order parameter values β have significant effects on the thermal quality factor in the context of the Lord–Shulman model. In the context of the two studied types of fractional derivative definition, an increase in the value β leads to an increase in the thermal quality factor’s value. Each curve has a peak point, which is in the following order:

(38) $$Q_{\mathrm{Max}}^{-1}\left(F-C,\;\beta =0.8\right)>Q_{\mathrm{Max}}^{-1}\left(F-C,\;\beta =0.3\right)>Q_{\mathrm{Max}}^{-1}\left(C-C,\;\beta =0.8\right)>Q_{\mathrm{Max}}^{-1}\left(C-C,\;\beta =0.3\right)$$

Figure 5 shows that the type of the fractional derivative and the fractional-order parameter values β have significant effects on the thermal quality factor in the context of the Tzou model. In the context of the classical Caputo fractional derivative definition, an increase in the value β leads to an increase in the thermal quality factor’s value. In the Caputo–Fabrizio model, an increase in the value β leads to a decrease in the thermal quality factor’s value. Each curve has a peak point, which is in the following order:

(39) $$Q_{\mathrm{Max}}^{-1}\left(F-C,\;\beta =0.8\right)>Q_{\mathrm{Max}}^{-1}\left(F-C,\;\beta =0.3\right)>Q_{\mathrm{Max}}^{-1}\left(C-C,\;\beta =0.3\right)>Q_{\mathrm{Max}}^{-1}\left(C-C,\;\beta =0.8\right)$$

**Figure 6:**
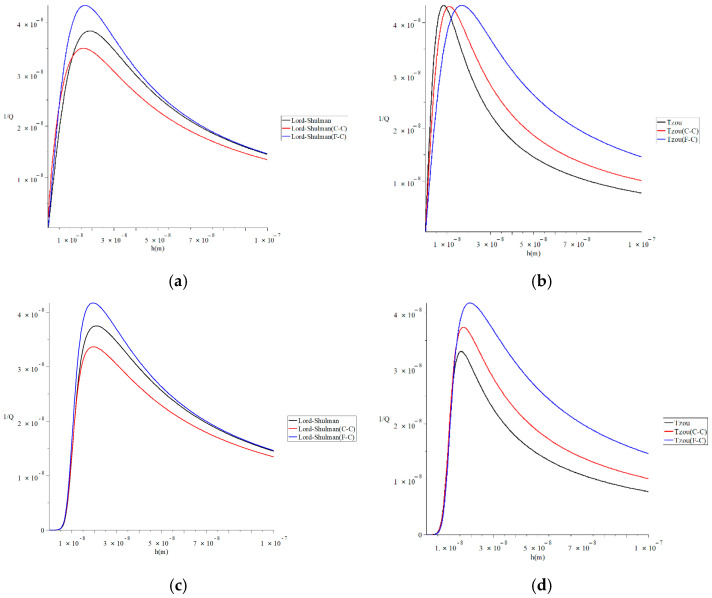
The thermal quality factor for different models with various values of the force due to static prestress when L=50 h,  b=4 h,  q=4.73/L,  and  β=0.6. (**a**) The thermal quality factor for the Lord–Shulman model when *F* = 0.0 *N*; (**b**) the thermal quality factor for the Tzou model when *F* = 0.0 *N*; (**c**) the thermal quality factor for the Lord-Shulman model when *F* = 10^−40^ *N*; (**d**) the thermal quality factor for the Tzou model when *F* = 10^−40^ *N.*

Figure 6 represents the thermal quality factor for different models with various values of the force due to static prestress $F=\left(0.0,10^{-40}\right)N$ when L=50 h, b=4h,  q=4.73/L and β=0.6 to stand on its effect on the thermal quality factor. This figure shows that the force’s value due to static prestress significantly impacts the thermal quality factor under the two studied models of heat conduction and the two studied definitions of a fractional derivative. Moreover, the force’s effects due to static prestress in the context of the Lord–Shulman model are not the same in the context of the Tzou model. To clarify that, we focus on the values of the peak points of the thermal quality factor, which take the following orders:

In Figure 6a, the maximum values of the quality factor take the following order:

(40) $$Q_{Max}^{-1}\left(L-S\left(F-C\right),\;F=0.0\right)>Q_{Max}^{-1}\left(L-S,\;F=0.0\right)>Q_{Max}^{-1}\left(L-S\left(C-C\right),\;F=0.0\right)$$

In Figure 6b, the maximum values of the quality factor take the following order:

(41) $$Q_{Max}^{-1}\left(Tzou\left(F-C\right),\;F=0.0\right)\approx Q_{Max}^{-1}\left(Tzou,\;F=0.0\right)\approx Q_{Max}^{-1}\left(Tzou\left(C-C\right),\;F=0.0\right)$$

In Figure 6c, the maximum values of the quality factor take the following order:

(42) $$Q_{Max}^{-1}\left(L-S\left(F-C\right),\;{F=10}^{-40}\right)>Q_{Max}^{-1}\left(L-S,\;{F=10}^{-40}\right)>Q_{Max}^{-1}\left(L-S\left(C-C\right),\;{F=10}^{-40}\right)$$

In Figure 6d, the maximum values of the quality factor take the following order:

(43) $$Q_{Max}^{-1}\left(L-S\left(F-C\right),\;{F=10}^{-40}\right)>Q_{Max}^{-1}\left(L-S\left(C-C\right),\;{F=10}^{-40}\right)>Q_{Max}^{-1}\left(L-S,\;{F=10}^{-40}\right)$$

**Figure 7:**
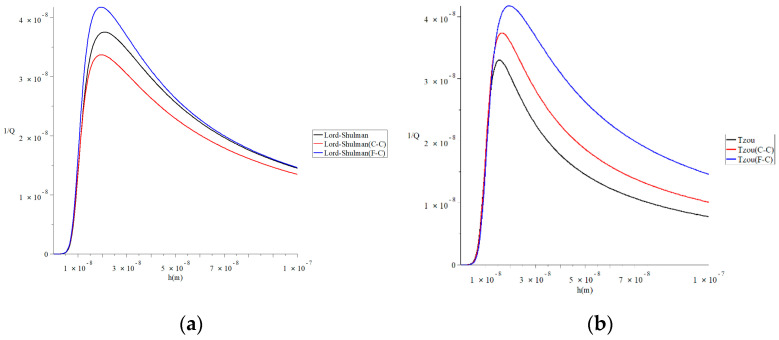

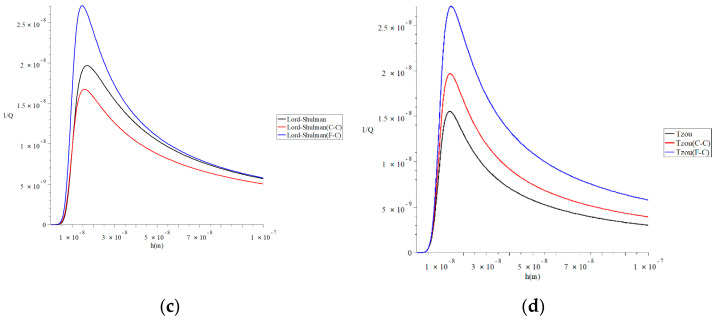
The thermal quality factor for different models with various isothermal values of natural frequency when $L=50\;h,\;\;b=4h,\;\;F=10^{-40}N,\;\;\mathrm{and}\;\;\beta =0.6$. (**a**) The thermal quality factor for the Lord–Shulman model when *q* = 4.73/*L*; (**b**) the thermal quality factor for the Tzou model when *q* = 4.73/*L*; (**c**) the thermal quality factor for the Lord-Shulman model when *q* = 7.85/*L*; (**d**) the thermal quality factor for the Tzou model when *q* = 7.85/*L*.

Figure 7 represents the thermal quality factor for different models with various isothermal values of natural frequency $\omega_{0}\left(q\right)=\omega_{0}\left(4.73/L,7.85/L\right)$ when L=50 h, b=4h, $F=10^{-40}N$ and β=0.6 to stand on its effect on the thermal quality factor. This figure shows that the isothermal value of natural frequency significantly impacts the thermal quality factor under the two studied heat conduction models and the two studied definitions of a fractional derivative. Moreover, the effects of the isothermal values of natural frequency in the Lord–Shulman model are not the same in the context of the Tzou model. To clarify that, we focus on the values of the peak points of the thermal quality factor, which take the following orders:

In Figure 7a, the maximum values of the quality factor take the following order:

(44) $$Q_{Max}^{-1}\left(L-S\left(F-C\right),\;q=4.73/L\right)>Q_{Max}^{-1}\left(L-S,\;q=4.73/L\right)>Q_{Max}^{-1}\left(L-S\left(C-C\right),\;q=4.73/L\right)$$

In Figure 7b, the maximum values of the quality factor take the following order:

(45) $$Q_{Max}^{-1}\left(Tzou\left(F-C\right),\;q=4.73/L\right)>Q_{Max}^{-1}\left(Tzou\left(C-C\right),\;q=4.73/L\right)>Q_{Max}^{-1}\left(Tzou,\;q=4.73/L\right)$$

In Figure 7c, the maximum values of the quality factor take the following order:

(46) $$Q_{Max}^{-1}\left(L-S\left(F-C\right),\;q=7.83/L\right)>Q_{Max}^{-1}\left(L-S,\;q=7.83/L\right)>Q_{Max}^{-1}\left(L-S\left(C-C\right),\;q=7.83/L\right)$$

In Figure 7d, the maximum values of the quality factor take the following order:

(47) $$Q_{Max}^{-1}\left(Tzou\left(F-C\right),\;q=7.85/L\right)>Q_{Max}^{-1}\left(Tzou\left(C-C\right),\;q=7.85/L\right)>Q_{Max}^{-1}\left(Tzou,\;q=7.85/L\right)$$

**Figure 8:**
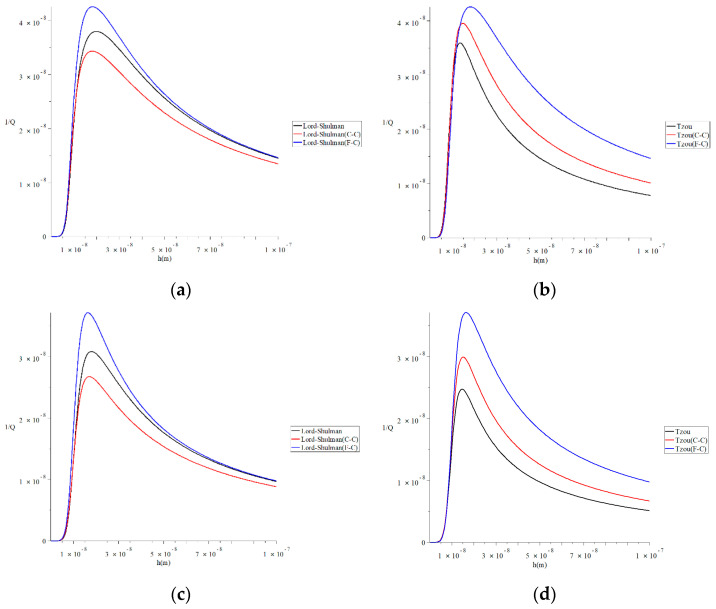
The thermal quality factor for different models with various values of the beam’s length when $q=4.73/L,\;\;b=4\;h,\;\;F=10^{-40}\;N,\;\;\mathrm{and}\;\;\beta =0.6$. (**a**) The thermal quality factor for the Lord–Shulman model when *L* = 30 h; (**b**) the thermal quality factor for the Tzou model when *L* = 30 h; (**c**) the thermal quality factor for the Lord-Shulman model when *L* = 50 h; (**d**) the thermal quality factor for the Tzou model when *L* = 50 h.

Figure 8 represents the thermal quality factor for different models with various values of the beam’s length L=(30,50)h when q=4.73/L b=4h, $F=10^{-40}N$, and β=0.6 to stand on its effect on the thermal quality factor. This figure shows that the value of the beam’s size significantly impacts the thermal quality factor under the two studied models of heat conduction and the two studied definitions of a fractional derivative. Moreover, the effects of the beam’s length in the Lord–Shulman model’s context are not the same in the context of the Tzou model. To clarify that, we focus on the values of the peak points of the thermal quality factor, which take the following orders.

In Figure 8a, the maximum values of the quality factor take the following order:

(48) $$Q_{Max}^{-1}\left(L-S\left(F-C\right),\;L=30\;h\right)>Q_{Max}^{-1}\left(L-S,\;L=30\;h\right)>Q_{\mathrm{Max}}^{-1}\left(L-S\left(C-C\right),\;L=30\;h\right)$$

In Figure 8b, the maximum values of the quality factor take the following order:

(49) $$Q_{Max}^{-1}\left(Tzou\left(F-C\right),\;L=30\;h\right)>Q_{Max}^{-1}\left(Tzou\left(C-C\right),\;L=30\;h\right)>Q_{Max}^{-1}\left(Tzou,\;L=30\;h\right)$$

In Figure 8c, the maximum values of the quality factor take the following order:

(50) $$Q_{Max}^{-1}\left(L-S\left(F-C\right),L=50\;h\right)>Q_{Max}^{-1}\left(L-S,\;L=50\;h\right)>Q_{Max}^{-1}\left(L-S\left(C-C\right),\;L=50\;h\right)$$

In Figure 8d, the maximum values of the quality factor take the following order:

(51) $$Q_{Max}^{-1}\left(Tzou\left(F-C\right),\;L=50\;h\right)>Q_{Max}^{-1}\left(Tzou\left(C-C\right),\;L=50\;h\right)>Q_{Max}^{-1}\left(Tzou,\;L=50\;h\right)$$

To verify the results, we take the value of the fractional-order parameter β=1, then the results in the current work are the same as [8,19,20,42,43,49,57,58].

## 5. Conclusions

A thermal study of the thermal quality factor was applied. A force attributable to static prestress was found to create a purely assisted microbeam resonator made of silicon (Si). The governing equations have been built in the context of the six different thermal conductivity models, the traditional Lord–Shulman, the classical Lord–Shulman based on Caputo, Lord–Shulman based on the Fabrizio–Caputo, the traditional Tzou, the classical Caputo, and the fractional Fabrizio–Caputo.

The findings demonstrate that the thermal quality factor’s intensity has significant consequences attributable to static prestress, fractional order parameters, natural frequency isothermal strength, and beam length.

The two fractional derivatives definitions influence the thermal quality factor differently and substantially.

The thermal quality factor obtains a maximum value in the context of the Tzou dual-phase lag model or Lord–Shulman model when it is based on the fractional Fabrizio–Caputo definition.

An increase in the fractional-order parameter leads to an increase in the thermal quality value of the nanobeam.
